# Supplementary material for: Microbial hydrocarbon degradation potential of the Baltic Sea ecosystem
Source: Microbiome. 2025 Oct 15;13:204. doi: 10.1186/s40168-025-02211-w (PMC12529799; doi:10.1186/s40168-025-02211-w)
Supplement: Supplementary file 2 — Additional file 1: Supplementary Fig. S1 Map of the sampling locations of the 203 Baltic Sea metagenomes. Colors indicate the distribution of each sample based on the Baltic Sea sub-basin category of HELCOM (2022). Supplementary Fig. S2 Histograms showing the distribution of MAG characteristics. (a) Genome size, (b) GC Content, (c) N50 (contigs), and (d) predicted gene counts. Colors represent the environment where the MAGs were located, i.e., pelagic (purple) and benthic (brown). The dashed line indicates mean values. Supplementary Fig. S3 Scatter plots of the relationship between HDG Count and Genome Size (bp) for (a) all MAGs, (b) benthic MAGs, and (c) pelagic MAGs. Supplementary Fig. S4 Frequency of HDGs annotated per Baltic Sea sub-basin. Supplementary Fig. S5 Alpha diversity estimates of the hydrocarbon degradation genes (HDGs) per sub-basin for (a) benthic and (b) pelagic samples. Supplementary Fig. S6 Upset plot of the shared and unique HMM annotations per Baltic Sea sub-basin. Supplementary Fig. S7 Maximum-likelihood phylogenomic tree of the archaeal MAGs. Supplementary Fig. S8 Relative abundance of the top 10 most abundant species based on the total number of medium-quality MAGs visualized (a) per environment and (b) per Baltic Sea sub-basin. Supplementary Fig. S9 Upset plot of the shared and unique species per Baltic Sea sub-basin. Supplementary Fig. S10 Bubble plot of the classified microbial taxa at the phylum-level and the occurrence of hydrocarbon degradation genes (HDGs) in each taxa. Supplementary Fig. S11 Random-forest classification of the relative abundance HDGs across the environments: (left) HDGs presented in descending order of importance, and the (right) relative abundance of HDGs enriched in each environment. Supplementary Fig. S12 Random-forest classification of the relative abundance of HDG substrates across the environments: (left) Substrates presented in descending order of importance, and the (right) relative abundance of HDG substrates [file 40168_2025_2211_MOESM1_ESM.docx]

# Supporting Information for

**Microbial hydrocarbon degradation potential of the Baltic Sea ecosystem**

### Authors & Affiliations

Joeselle M. Serrana^1,2^*, Benoît Dessirier^1,3^, Francisco J. A. Nascimento^1,4^, Elias Broman^1,3,4^, and Malte Posselt^1,2^

^1^ Stockholm University Center for Circular and Sustainable Systems (SUCCeSS), Stockholm University, 106 91 Stockholm, Sweden

^2^ Department of Environmental Science (ACES), Stockholm University, 106 91 Stockholm, Sweden

^3^ Baltic Sea Centre, Stockholm University, Stockholm, Sweden

^4^ Department of Ecology, Environment, and Plant Sciences (DEEP), Stockholm University, 106 91 Stockholm, Sweden

*Correspondence: [joeselle.serrana@aces.su.se](mailto:joeselle.serrana@aces.su.se)

# Supplementary Table Legends

#### Supplementary Table S1. Metagenomics sample information and environmental factors. Metagenomics sequencing data were obtained from the European Nucleotide Archive (ENA) database: accession numbers PRJEB41834 (Broman et al., 2022; Rodríguez-Gijón et al., 2023), PRJEB22997 (Alneberg et al., 2018), and PRJEB34883 (Alneberg et al., 2020). The environmental parameters, i.e., depth (m), salinity (PSU), and temperature (°C), compiled by Rodríguez-Gijón et al. (2023) were used in this study.

#### Supplementary Table S2. Metagenomics data processing information: Sequence codes, read processing counts (from quality-filtering to contig assembly), prodigal annotations and binning stat values. The Calgary approach to ANnoTating HYDrocarbon degradation genes (CANT-HYD; Khot et al., 2022) counts highlighted in blue.

#### Supplementary Table S3. Metagenome-assembled genome (MAG) stats and taxonomic classification based on using GTDB-Tk v2.4.0 with R220 (Chaumeil et al., 2022).

#### Supplementary Table S4. The Calgary approach to ANnoTating HYDrocarbon degrading enzymes database (CANT-HYD) annotations (Khot et al. 2022).

#### Supplementary Table S5. Count and annotation table of the annotated hydrocarbon degradation genes (HDGs). Reads per kilobase million (RPKM) absolute counts.

#### Supplementary Table S6. The annual average of total oil spills during the assessment period 2016-2021 (m³) values (Supplementary Table S6) from HELCOM (2023b).

#### Supplementary Table S7. Average metagenome-assembled genome (MAG) quality stats per environment.

#### Supplementary Table S8. Total metagenome-assembled genome (MAG) counts per environment and relative abundance based on species-level classification.

#### Supplementary Table S9. Statistical results of the permutation test for homogeneity of multivariate dispersions and permutation test for adonis under a reduced model of the hydrocarbon degradation genes (HDG) dataset.

#### Supplementary Table S10. Differential abundance test by Random Forests for the HDGs per environment and per subbasin.

#### Supplementary Table S11. Statistical results of the permutation test for distance-based redundancy analysis (dbRDA) analyses in R of the ARG composition and environmental factors.

#### Supplementary Table S12. Spearman rank correlations between environmental variables and the RPKM abundance of the hydrocarbon degradation genes (HDG).

# Supplementary Figures


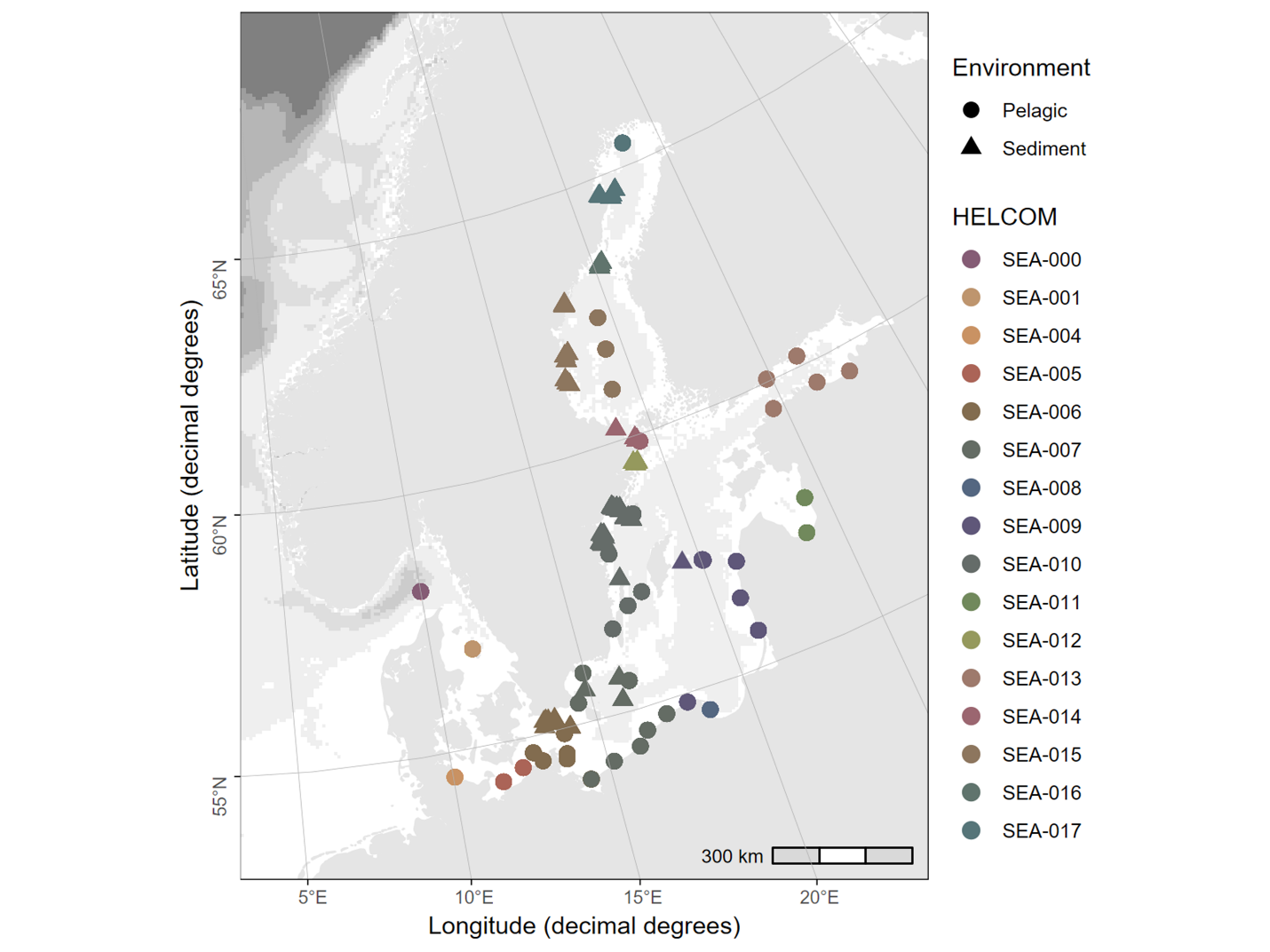


#### Supplementary Fig. S1. Map of the sampling locations of the 203 Baltic Sea metagenomes. Colors indicate the distribution of each sample based on the Baltic Sea sub-basin category of HELCOM (2022).


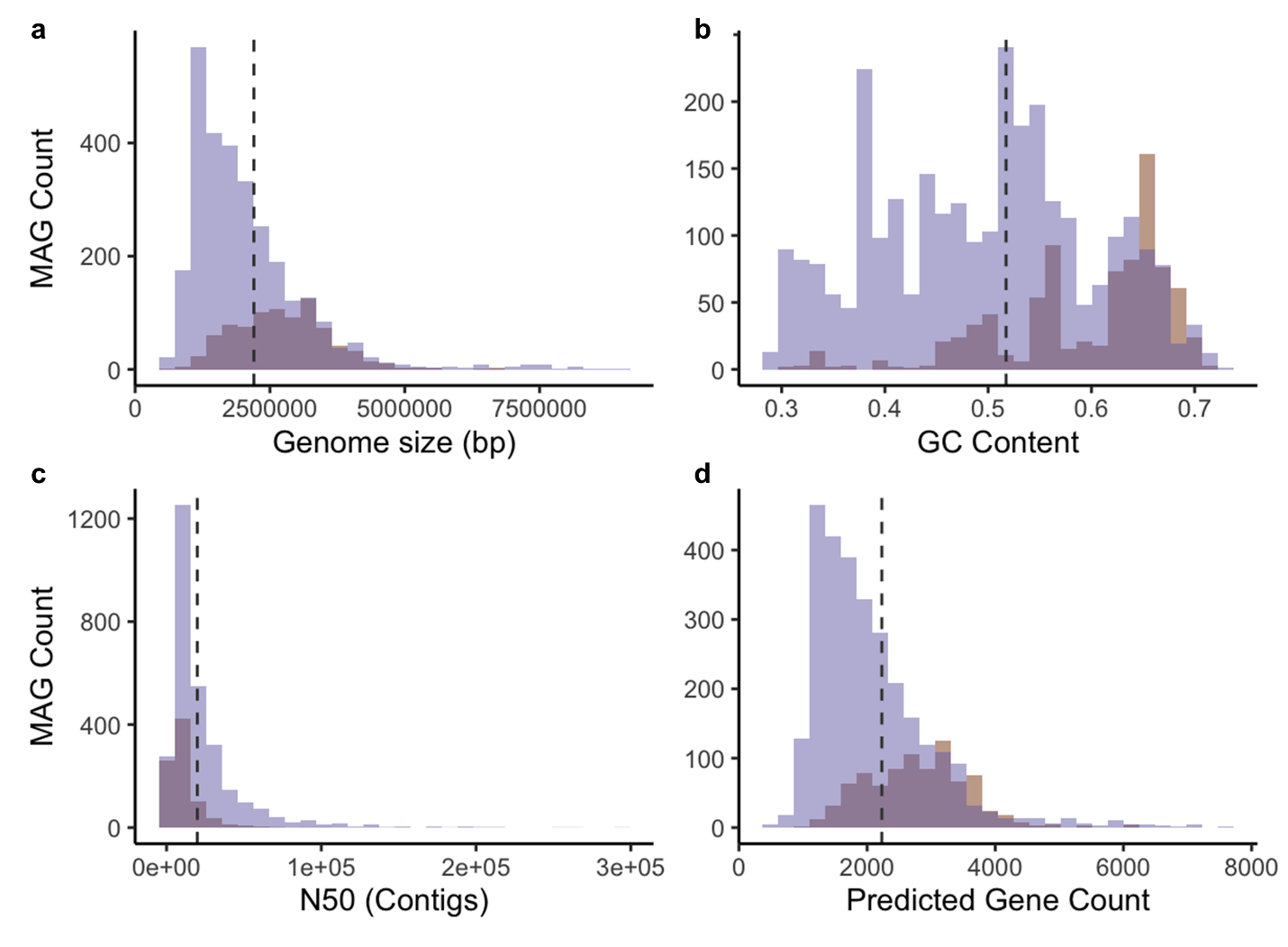


#### Supplementary Fig. S2. Histograms showing the distribution of MAG characteristics. (a) Genome size, (b) GC Content, (c) N50 (contigs), and (d) predicted gene counts. Colors represent the environment where the MAGs were located, i.e., pelagic (purple) and benthic (brown). The dashed line indicates mean values.


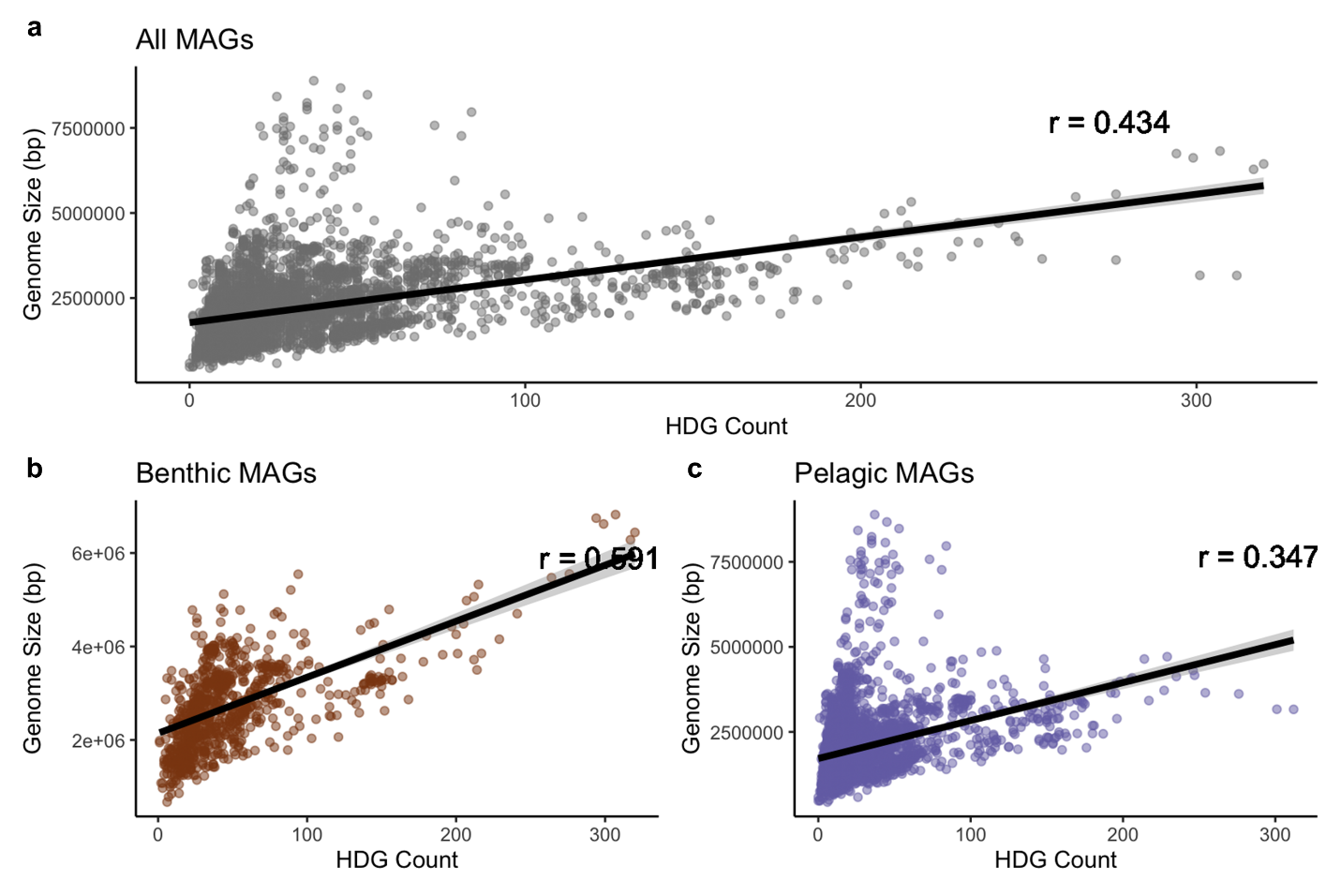


#### Supplementary Fig. S3. Scatter plots of the relationship between HDG Count and Genome Size (bp) for (a) all MAGs, (b) benthic MAGs, and (c) pelagic MAGs.


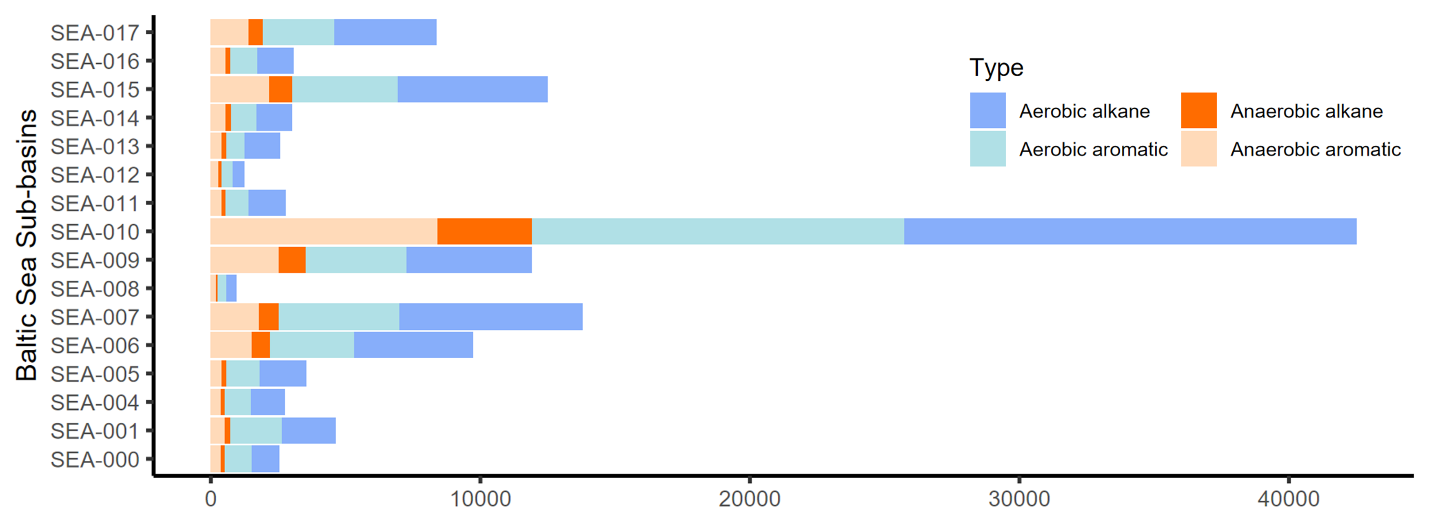


#### Supplementary Fig. S4. Frequency of HDGs annotated per Baltic Sea sub-basin.


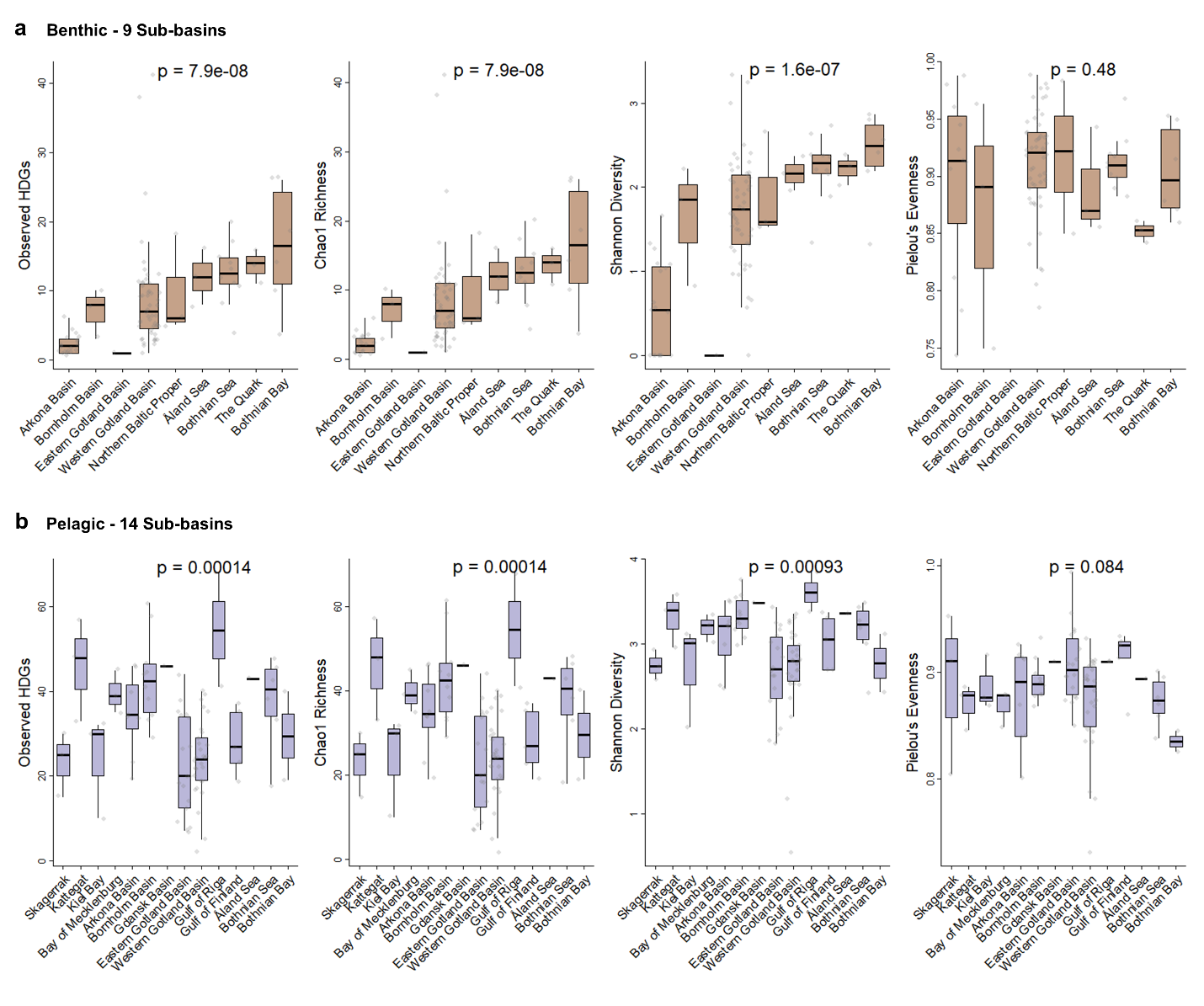


#### Supplementary Fig. S5. Alpha diversity estimates of the hydrocarbon degradation genes (HDGs) per sub-basin for (a) benthic and (b) pelagic samples.


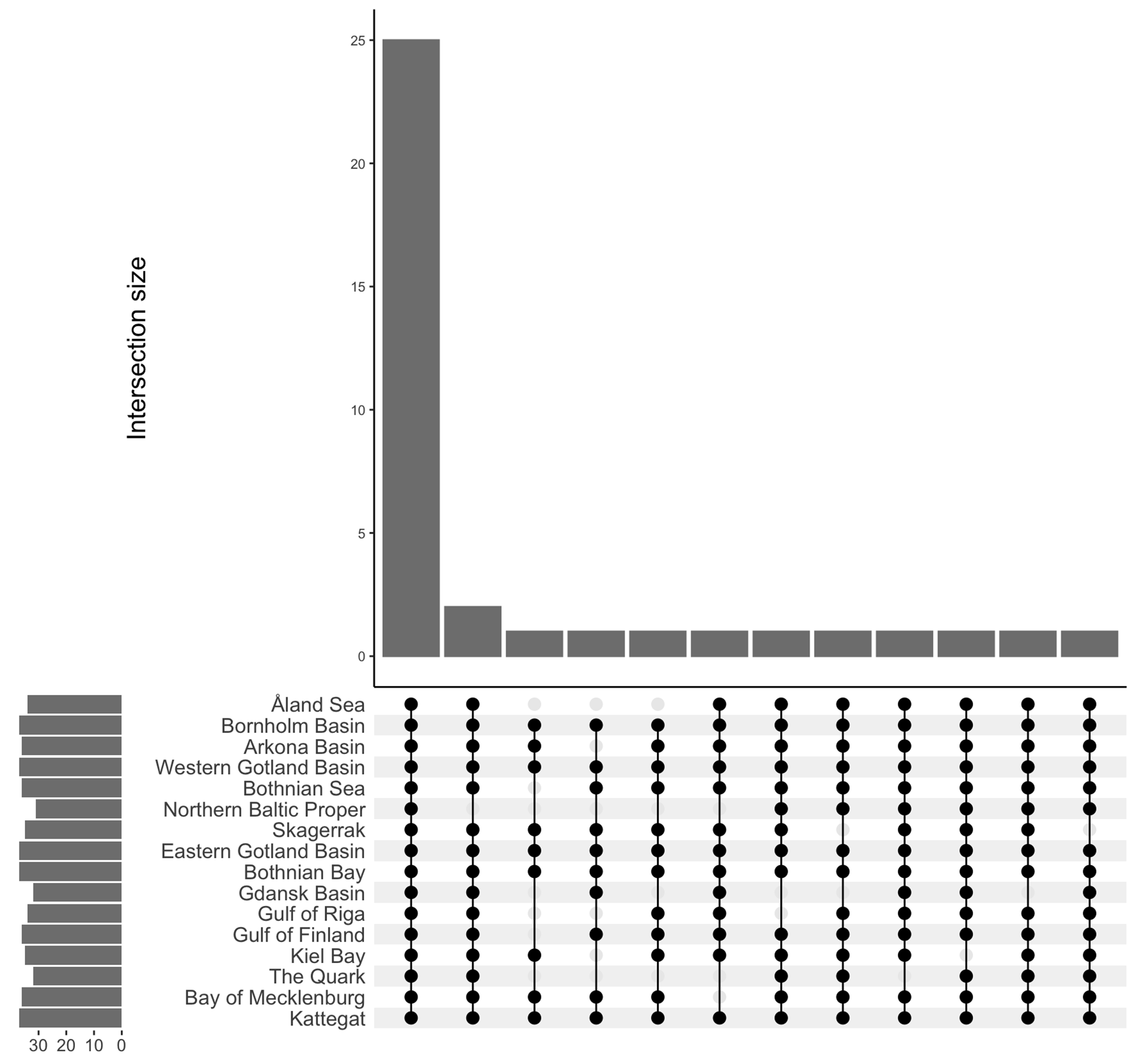


#### Supplementary Fig. S6. Upset plot of the shared and unique HMM annotations per Baltic Sea sub-basin.


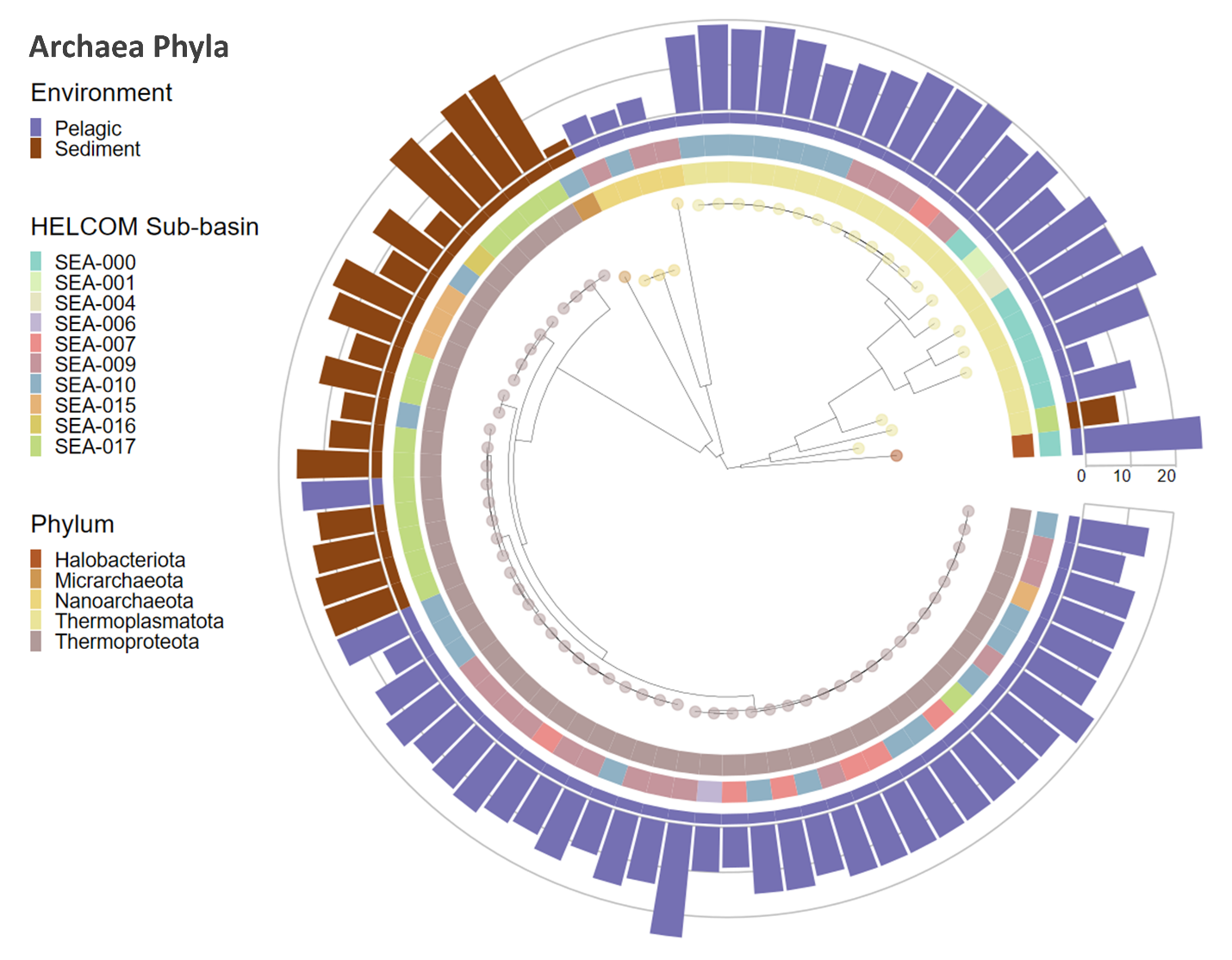


#### Supplementary Fig. S7. Maximum-likelihood phylogenomic tree of the archaeal MAGs.


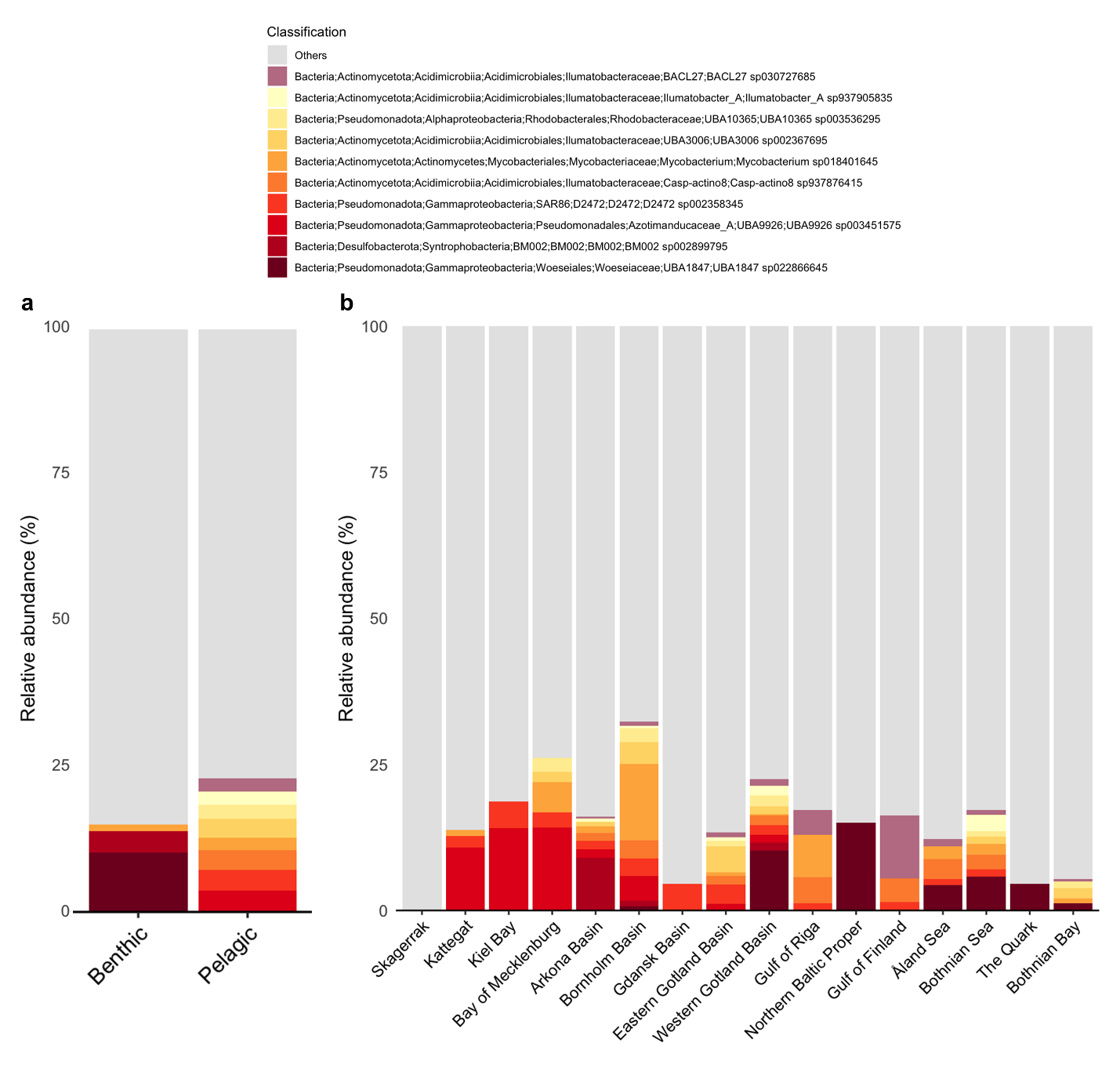


#### Supplementary Fig. S8. Relative abundance of the top 10 most abundant species based on the total number of medium-quality MAGs visualized (a) per environment and (b) per Baltic Sea sub-basin.


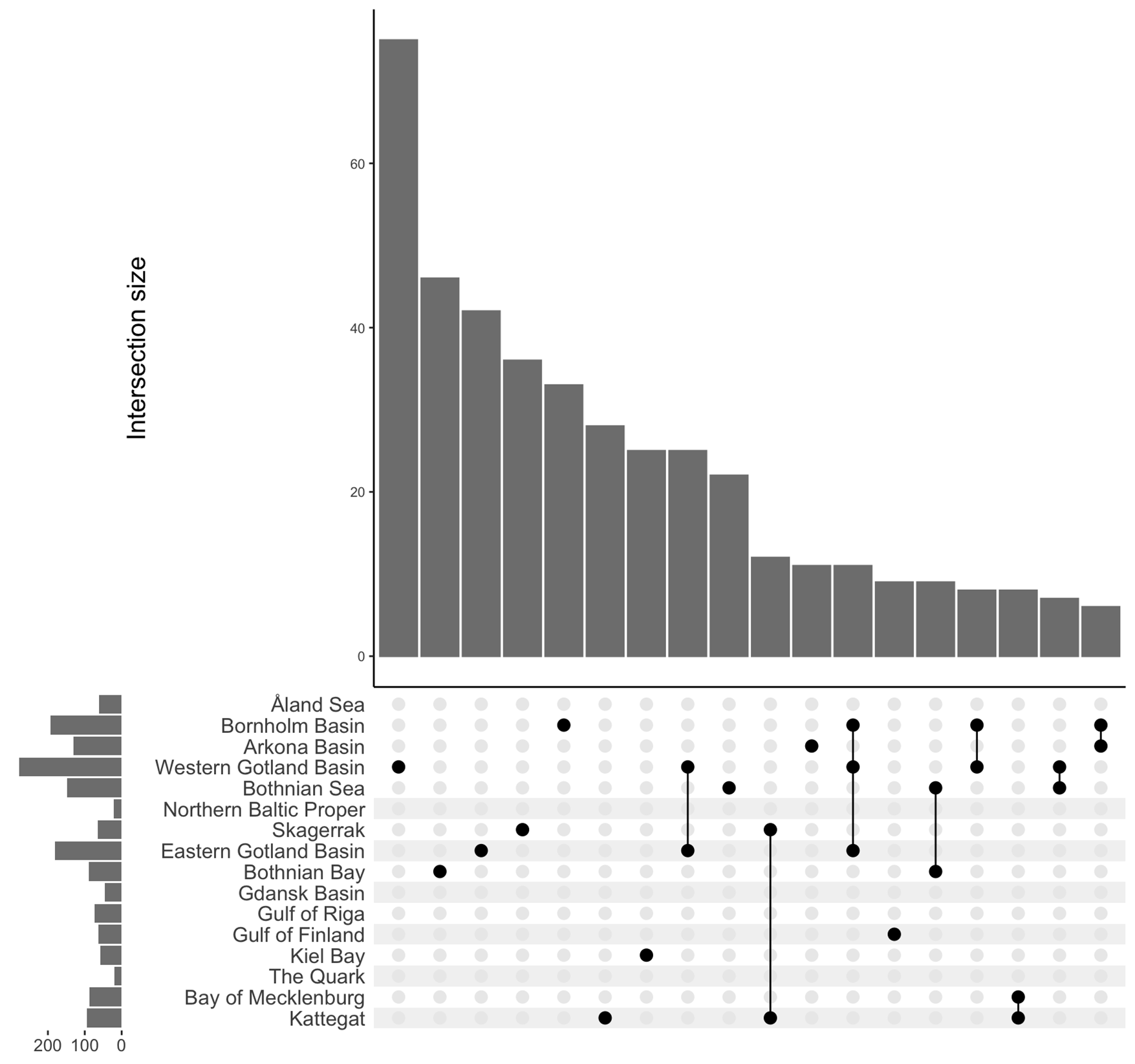


#### Supplementary Fig. S9. Upset plot of the shared and unique species per Baltic Sea sub-basin.


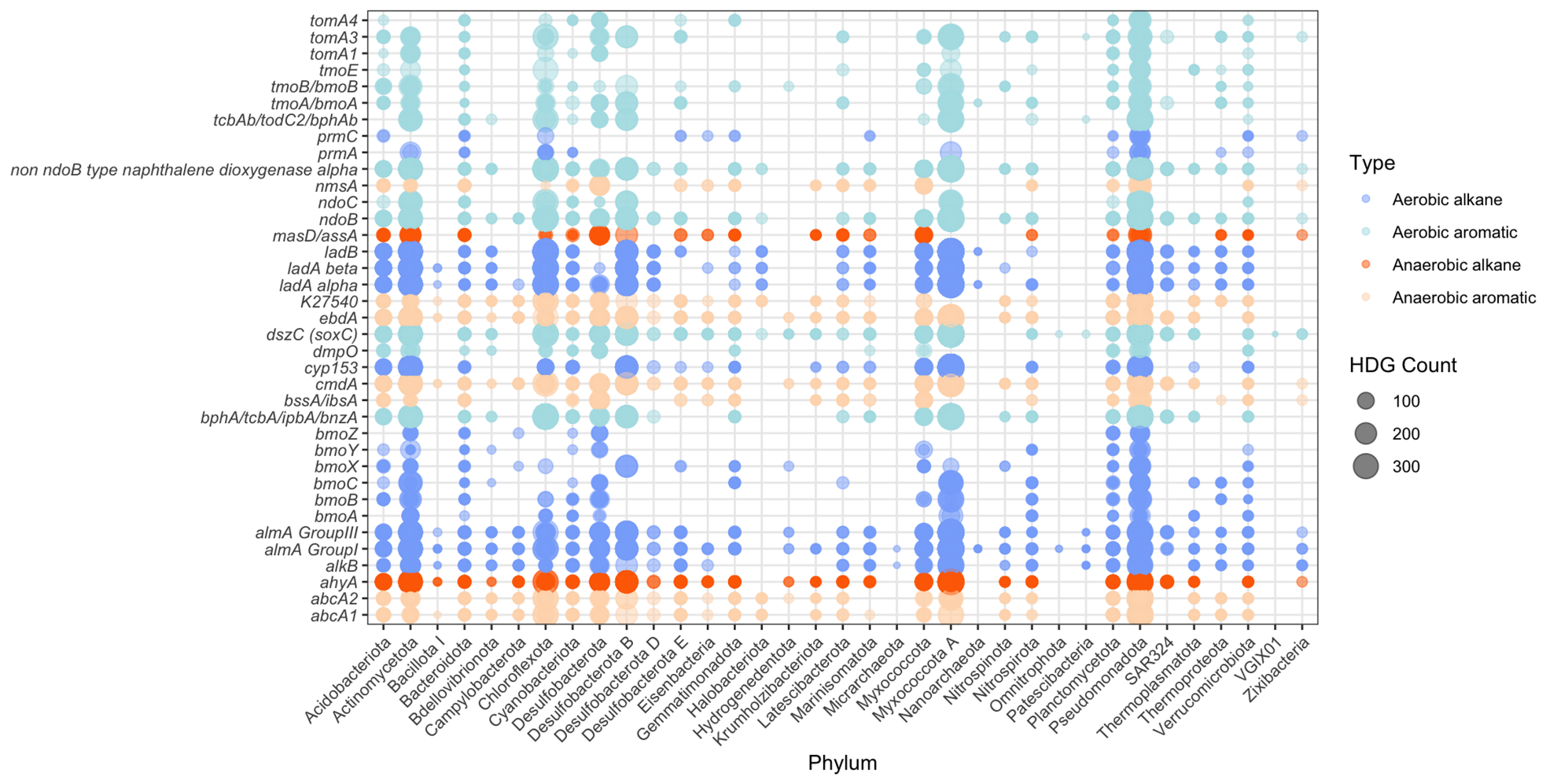


#### Supplementary Fig. S10. Bubble plot of the classified microbial taxa at the phylum-level and the occurrence of hydrocarbon degradation genes (HDGs) in each taxa.


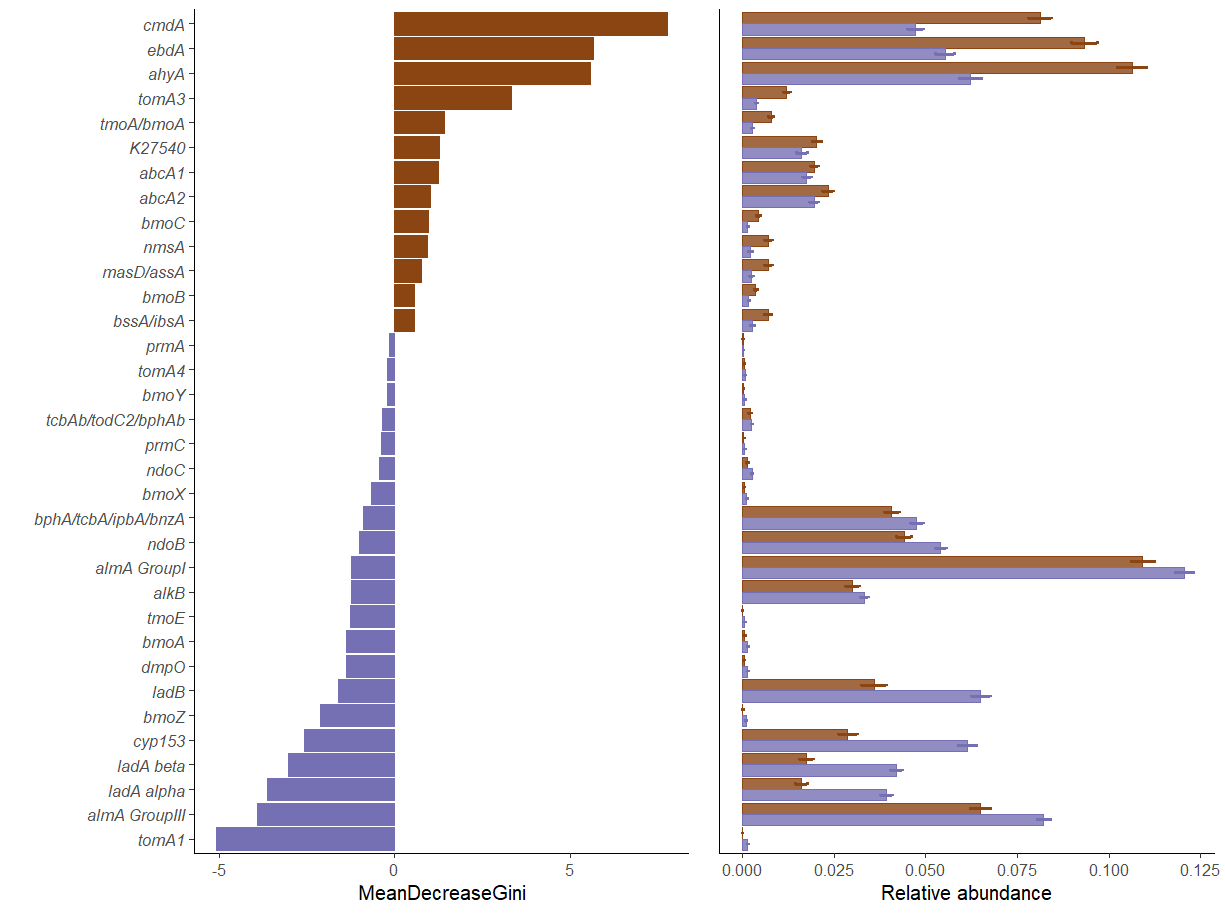


#### Supplementary Fig. S11. Random-forest classification of the relative abundance HDGs across the environments: (left) HDGs presented in descending order of importance, and the (right) relative abundance of HDGs enriched in each environment.


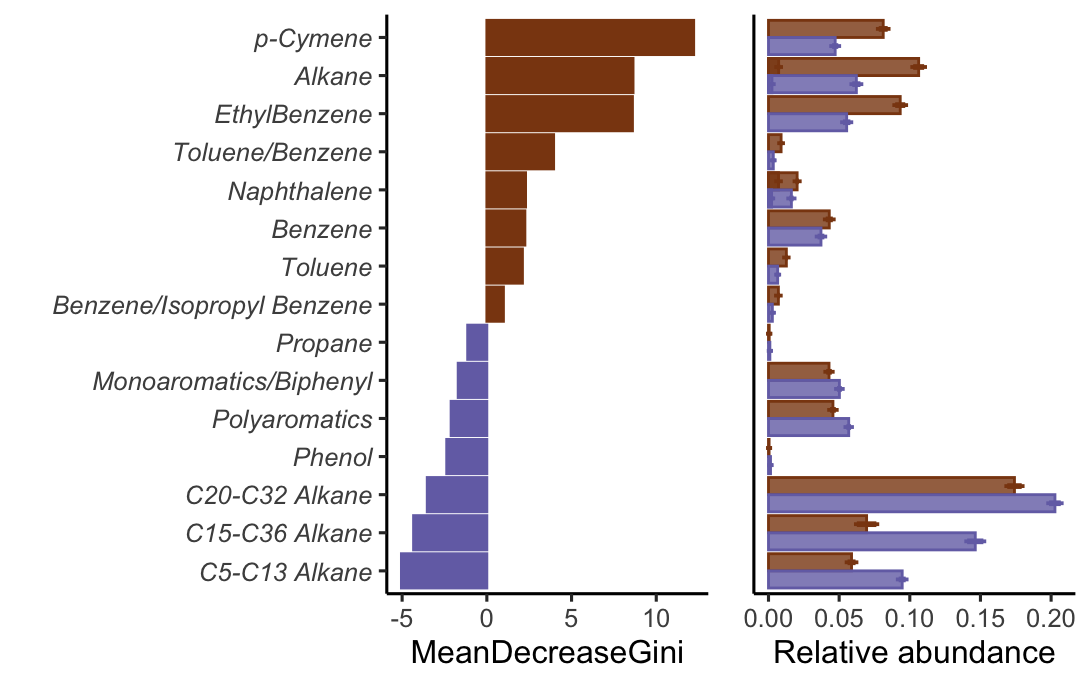


#### Supplementary Fig. S12. Random-forest classification of the relative abundance of HDG substrates across the environments: (left) Substrates presented in descending order of importance, and the (right) relative abundance of HDG substrates enriched in each environment.

## References:

Alneberg, J., Bennke, C., Beier, S., Bunse, C., Quince, C., Ininbergs, K., ... & Andersson, A. F. (2020). Ecosystem-wide metagenomic binning enables prediction of ecological niches from genomes. Communications Biology, 3(1), 119.

Alneberg, J., Sundh, J., Bennke, C., Beier, S., Lundin, D., Hugerth, L. W., ... & Andersson, A. F. (2018). BARM and BalticMicrobeDB, a reference metagenome and interface to meta-omic data for the Baltic Sea, Scientific Data, 5, 180146.

Broman, E., Izabel-Shen, D., Rodríguez-Gijón, A., Bonaglia, S., Garcia, S. L., & Nascimento, F. J. (2022). Microbial functional genes are driven by gradients in sediment stoichiometry, oxygen, and salinity across the Baltic benthic ecosystem. Microbiome, 10(1), 126.

Chaumeil, P. A., Mussig, A. J., Hugenholtz, P., & Parks, D. H. (2022). GTDB-Tk v2: memory friendly classification with the genome taxonomy database. Bioinformatics, 38(23), 5315-5316.

Khot, V., Zorz, J., Gittins, D. A., Chakraborty, A., Bell, E., Bautista, M. A., ... & Bhatnagar, S. (2022). CANT-HYD: a curated database of phylogeny-derived hidden Markov models for annotation of marker genes involved in hydrocarbon degradation. Frontiers in Microbiology, 12, 764058.

HELCOM (2022). HELCOM Monitoring and Assessment Strategy: Attachment 4 - HELCOM sub-divisions of the Baltic Sea, 23-39.

HELCOM (2023a). HELCOM Thematic assessment of hazardous substances, marine litter, underwater noise and non-indigenous species 2016-2021. Baltic Sea Environment Proceedings 190. https://helcom.fi/post_type_publ/holas3_haz/

Rodríguez-Gijón, A., Buck, M., Andersson, A. F., Izabel-Shen, D., Nascimento, F. J., & Garcia, S. L. (2023). Linking prokaryotic genome size variation to metabolic potential and environment. ISME Communications, 3(1), 25.
